# Innovative application of ceftriaxone as a quorum sensing inhibitor in *Pseudomonas aeruginosa*

**DOI:** 10.1038/s41598-025-87609-0

**Published:** 2025-02-11

**Authors:** Nourhan G. Naga, Dalia E. El-Badan, Mona E. M. Mabrouk, Heba S. Rateb, Khaled M. Ghanem, Mona I. Shaaban

**Affiliations:** 1 Department of Botany and Microbiology, Faculty of Science, Alexandria University, Alexandria, Egypt; 2 Department of Biological Sciences, Faculty of Science, Beirut Arab University, Beirut, Lebanon; 3 Botany and Microbiology Department, Faculty of Science, Damanhour University, Damanhour, Egypt; 4 Department of Pharmaceutical and Medicinal Chemistry, Pharmacy College, Misr University for Science and Technology, Cairo, Egypt; 5 Department of Microbiology and Immunology, Faculty of Pharmacy, Mansoura University, Mansoura, Egypt

**Keywords:** Quorum sensing inhibition, Ceftriaxone, *Pseudomonas aeruginosa*, Virulence factors, Ceftriaxone derivatives, Biotechnology, Microbiology

## Abstract

*Pseudomonas aeruginosa* is a Gram-negative bacterium that has the potential to induce various healthcare-related infections through its array of virulence factors. The control of virulence factor expression is mainly regulated by a communication process among cells called quorum sensing (QS). Blocking QS could be a viable tactic to suppress virulence factors and reduce pathogenicity without impacting bacterial growth. This approach has the potential to significantly decrease the multiple drug resistance emergence. In this study, we explored the impact of ceftriaxone (CRO), which is a commonly used β-lactam antibiotic, and its metal derivatives on the QS system and virulence factors of both standard strains and clinical isolates of *P. aeruginosa*. The quorum sensing inhibitory (QSI) activity of CRO and ceftriaxone Nickel complex (CRON) was evaluated. The minimum inhibitory concentration (MIC) was determined and the effect of sub-MICs of CRO and CRON was assessed on *P. aeruginosa* strains virulence factors. CRO and CRON effectively suppressed the virulence factors of *P. aeruginosa* strains at sub-MICs, without altering bacterial viability. Additionally, a molecular docking investigation was carried out to identify potential mechanisms of QSI. CRO and CRON exhibited high ICM scores, potentially displacing natural ligands when interacting with LasR, LasI, and PqsR receptors.

**Supplementary Information:**

The online version contains supplementary material available at 10.1038/s41598-025-87609-0.

## Introduction

*Pseudomonas aeruginosa* is a Gram-negative bacterial pathogen that causes a variety of diseases to many vital organs including the eyes, lungs, urinary tract, burn wounds, and respiratory tract^1–3^. *P. aeruginosa* uses multiple virulence factors, including motility, proteases, and toxins to produce infection^4^. *P. aeruginosa* poses a significant worldwide health risk due to its resistance to antibiotics and disinfectants^5^. The Centers for Disease Control and Prevention (CDC) has recently highlighted the ongoing threat of multiple-drug-resistant (MDR) *P. aeruginosa*, which was responsible for approximately 32,600 infections in hospitalized patients and 2,700 deaths in the United States in 2017, according to a 2019 CDC report on antibiotic resistance threats^6^. *P. aeruginosa* produces biofilms during infection, increasing resistance to drugs despite being sensitive when cultured planktonically^7^. The intrinsic resistance of *P. aeruginosa* is mostly due to reduced membrane permeability and multidrug efflux pumps^8,9^. Bacterial pathogens, such as *P. aeruginosa* use quorum-sensing (QS) systems to control their pathogenicity and virulence factors production^10^. The pathogenicity of *P. aeruginosa* is controlled by three QS systems. lasI/lasR, rhlI/rhlR, and *Pseudomonas* quinolone signal^11–14^. The QS systems in *P. aeruginosa* are structured hierarchically and governed by the LasR/LasI system. This system generates and detects the signaling molecule N-3-oxo-dodecanoyl-L-homoserine lactone triggering the synthesis of virulence factors like protease, pyocyanin, and elastase. The LasR/LasI system then activates the RhlR/RhlI QS system, which controls the secretion and detection of the autoinducer, N-butyryl-L-homoserine lactone. This mechanism regulates rhamnolipids and pyocyanin levels^15,16^. QS inhibition may reduce virulence and offer an alternative to conventional antibiotic treatments^17^. To ensure a successful antimicrobial therapy, bacterial resistance to antibiotics is a concerning issue that needs to be resolved, particularly in aggressive infections like those seen in *P. aeruginosa* infections. Several strategies have been put out to combat bacterial resistance^18^. A potential tactic that gives the immune system a chance to get rid of the bacterial invaders is to target the pathogenicity of the bacteria. Targeting QS to reduce bacterial pathogenesis is a sensible approach since QS serves as the virulence regulator that controls bacterial pathogenesis. Many safe and well-known prescribed drugs received particular attention as they have shown the ability to quench bacterial QS^19^. Over the past few years, there has been a rise in interest in using approved antibiotics for targeting bacterial pathogenicity and QS. For example, the QSI activity of doxycycline, β-lactams, cefoperazone, imipenem, and ceftazidime have exhibited QSI activity at sub-MICs^20–23^.

Ceftriaxone (CRO) is a third-generation cephalosporin with broad-spectrum bactericidal properties, primarily effective against Gram-negative bacteria. However, due to the emergence of resistance, CRO has become ineffective in treating *Pseudomonas* infections. To address this challenge, there is a need for novel approaches that target virulence factors without promoting resistance. Previous research by^24^ has explored metallic derivatives of CRO with antibacterial activity against Gram-negative bacilli. However, the QSI activity of CRO and its metallic derivatives remains unexplored. This research seeks to explore the effects of CRO and its metallic derivatives on virulence factors and QS pathways as a possible new approach to treating *Pseudomonas* infections.

## Results

### Assessment of QSI for metallic-ceftriaxone compounds with *C. Violaceum* ATCC 12,472

The violacein pigment production was inhibited by CRO, CRON, CROZ, and CROU by 22, 23, 15, and 9 mm. While CROD did not show any QSI activity. Both CRO and CRON exhibited the highest elimination in the violacein pigment production. Consequently, the effects of CRO and CRON were chosen to look into how they affected the virulence factors of both standard strains and clinical isolates of *P. aeruginosa*.

### Determination of minimum inhibitory concentration

The minimum inhibitory concentration of CRO and CRON against PA14, PAO1, Pn11, Pn13, Pn15, and Pn17 were assessed and indicated in Table 1. All further studies were conducted at the subinhibitory concentration (1/2 MIC and 1/4 MIC) to assess the anti-virulence and anti-QS properties of CRO and CRON.

### Effect of CRO and CRON sub‑MICs on the growth of *P. aeruginosa* strains

**Fig. 1:**
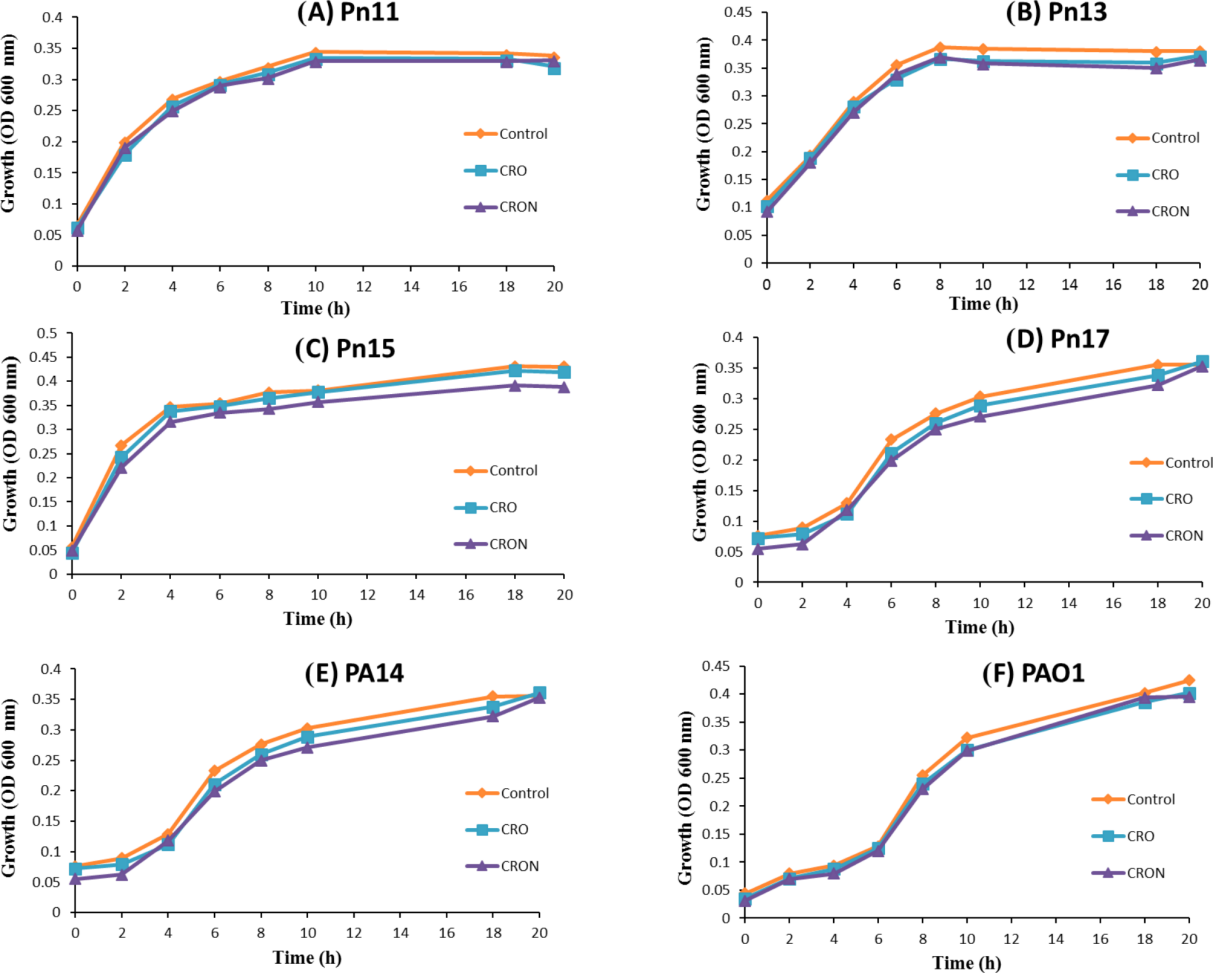
Growth curves of *P. aeruginosa* strains with and without 1/2 MIC of CRO and CRON.

The antibacterial impact of CRO and CRON on *P. aeruginosa* strains was assessed both with and without adding the tested compounds at 1/2 MIC to the growing cells for 24 h at 37 °C. The bacterial count of the untreated cultures was 111, 94, 98, 113, 122, and 116 × 10^7^ CFU/mL PA14, PAO1, Pn11, Pn13, Pn15, and Pn17, respectively. While the count of the treated cultures was 110, 92, 96, 110, 121, and 115 × 10^7^ CFU/mL for PA14, PAO1, Pn11, Pn13, Pn15, and Pn17, respectively. The number of bacteria in the treated and untreated cultures did not differ significantly. Moreover, there was no difference in the rate of bacterial growth between cultures treated with 1/2 MIC of CRO and CRON and those that were left untreated (Fig. 1).

### Impact of Sub-MIC levels of CRO and CRON on the virulence of *P. aeruginosa* strains

At 1/2 and 1/4 MICs, CRO and CRON significantly inhibited hemolysin, biofilm formation, protease activity, and pyocyanin production in cultures of PA14, PAO1, Pn11, Pn13, Pn15, and Pn17, in comparison to untreated controls.

### Effect on biofilm formation

**Fig. 2:**
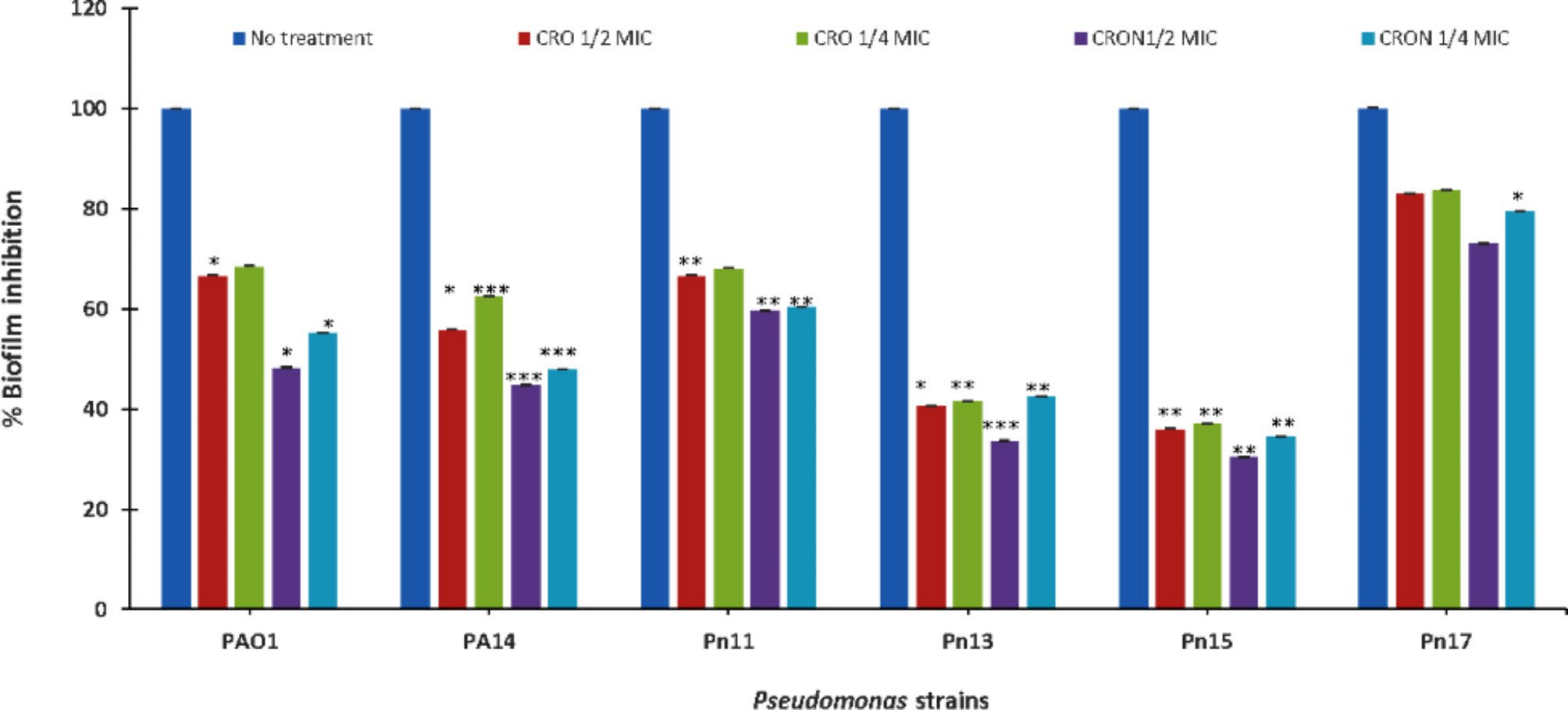
Effect of sub-MICs of CRO and CRON on biofilm formation of *P. aeruginosa* strains.

The biofilm development of PA14, PAO1, Pn11, Pn13, Pn15, and Pn17 was suppressed by using 1/2 MIC of CRO by 35%, 34%, 34%, 60%, 64%, and 17%. Biofilm was removed by treatments with 1/4 MIC of CRO by 32%, 38%, 32%, 59%, 63%, and 16%. Remarkably, CRON at 1/2 MIC considerably decreased the growth of biofilms by 55%, 52%, 40%, 66%, 70%, and 27%, in PA14, PAO1, Pn11, Pn13, Pn15, and Pn17, respectively. In the same instance, CRON at 1/4 MIC dramatically decreased biofilm by 45%, 52%, 39%, 58%, 66%, and 21%, respectively (Fig. 2).

### Effect on hemolysin production

**Fig. 3:**
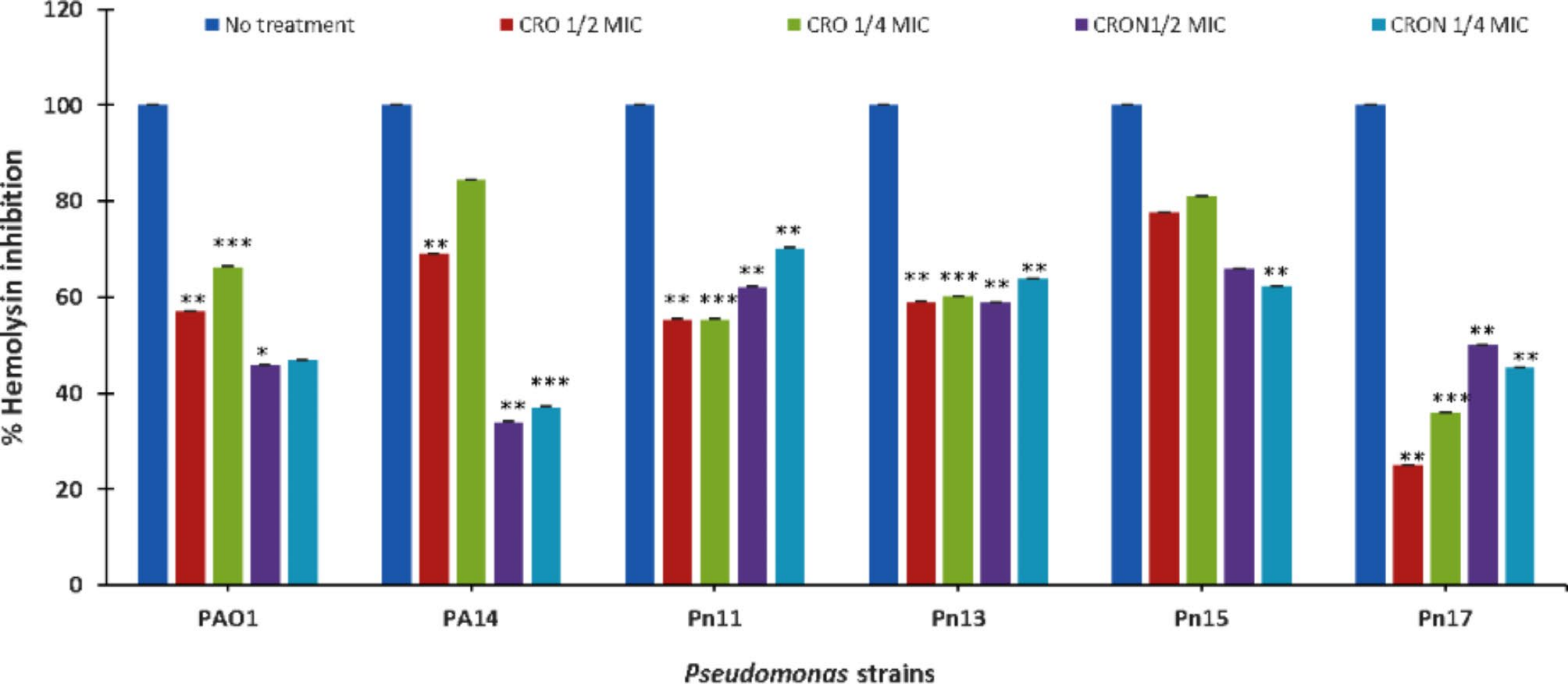
Effect of sub-MICs of CRO and CRON on hemolysin activity of *P. aeruginosa* strains.

Sub-MICs of CRO reduced hemolysin production significantly (*P* ≤ 0.01) in PA14, PAO1, Pn11, Pn13, Pn15, and Pn17 by 31%, 43%, 45%, 42%, 22%, and 75%, respectively at 1/2 MIC. While adding CRO at 1/4 MIC lessened hemolysin by 16%, 34%, 45%, 40%, 19%, 65%, and 34%, 16% in PA14, PAO1, Pn11, Pn13, Pn15, Pn17, and PA14, respectively. CRON at 1/2 MIC exhibited inhibition in hemolysin activity by 66%, 54%, 38%, 41%, 34%, and 50% in PA14, PAO1, Pn11, Pn13, Pn15, and Pn17, respectively. Likewise, 1/4 MIC of CRON diminished hemolysin activity by 30%, 53%, 36%, 38%, 55%, and 63% in PA14, PAO1, Pn11, Pn13, Pn15, and Pn17, respectively (Fig. 3).

### Effect of protease

**Fig. 4:**
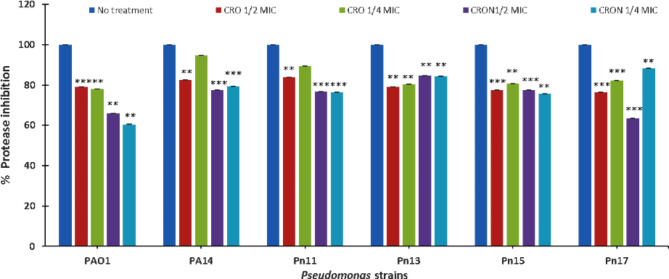
Effect of sub-MICs of CRO and CRON on protease activity of *P. aeruginosa* strains.

CRO at 1/2 MIC inhibited the protease activity significantly (*P* ≤ 0.01) by 18%, 21%, 16%, 21%, 23%, and 24% in PA14, PAO1, Pn11, Pn13, Pn15, and Pn17, respectively. *Pseudomonas* strains PA14, PAO1, Pn11, Pn13, Pn15, and Pn17 provided with 1/4 MIC reduced protease activity by 5%, 22%, 11%, 20%, 20%, and 18%, respectively. CRON at 1/2 MIC reduced the proteolytic activity significantly (*P* ≤ 0.001) by 23%, 34%, 23%, 15%, 22%, and 37% in PA14, PAO1, Pn11, Pn13, Pn15, and Pn17, respectively. Additionally, 1/4 MIC of CRON lessened protease by 21%, 40%, 23%, 15%, 24%, and 12% in PA14, PAO1, Pn11, Pn13, Pn15, and Pn17, respectively (Fig. 4).

### Effect on pyocyanin

**Fig. 5:**
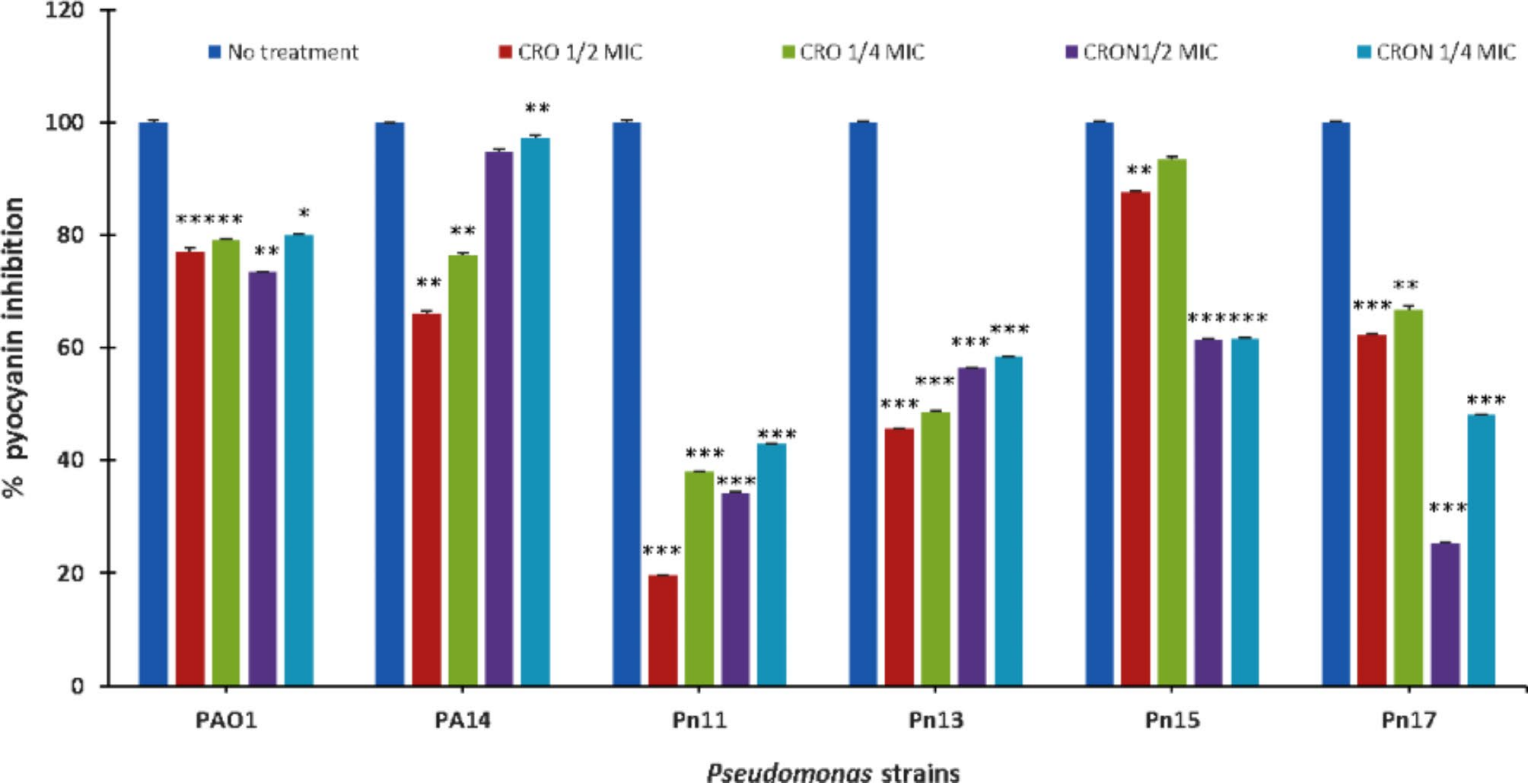
Effect of sub-MICs of CRO and CRON on pyocyanin production of *P. aeruginosa* strains.

Pyocyanin production was significantly (*P* ≤ 0.001) suppressed by CRO at 1/2 MIC in *P. aeruginosa* strains.; PA14, PAO1, Pn11, Pn13, Pn15, and Pn17 by 24%, 21%, 62%, 52%, 7%, and 33%, respectively. Moreover, 1/4 MIC of CRO decreased pyocyanin by 99%, 97%, 79%, 87%, and 99% in PA14, PAO1, Pn11, Pn13, Pn15, and Pn17, respectively. Interestingly, CRON at extremely low doses (1–16 µg/mL) decreased pyocyanin synthesis significantly (*P* ≤ 0.001) by 5%, 27%, 66%, 44%, 39%, and 75% in PA14, PAO1, Pn11, Pn13, Pn15, and Pn17, respectively. Additionally, pyocyanin levels were significantly decreased by 1/4 MIC of CRON by 3%, 20%, 57%, 42%, 38%, and 52% in PA14, PAO1, Pn11, Pn13, Pn15, and Pn17, respectively (Fig. 5).

### Expression of QS-regulated genes

The expression of *lasR* and *rhlI* in the untreated PAO1 was detected with Ct values (26.6 ± 0.01 and 31.6 ± 0.01, respectively. However, PAO1 treated with 1/2 of CORN showed extremely low and could not be properly measured (Supplementary Figs. 1 and 2). Henec, the relative expression of *lasR* and *rhlI* genes in the treated cells could not be determined as there is not obtained *Ct* values to be normalized to the average relative amount of *ropD* reference gene for treated isolate (Supplementary Figs. 1 and 2). The melting curve of *lasR* and *rhlI* indicated that the untreated and the treated isolates had the same melting profile without primer dimer and the formation of pure amplicons of the tested genes in untreated isolates. However, for the treated isolates there is no amplicon formed so the melting curve is very low.

### Docking

#### LasR and ligand binding affinity analysis using molecular docking

**Fig. 6:**
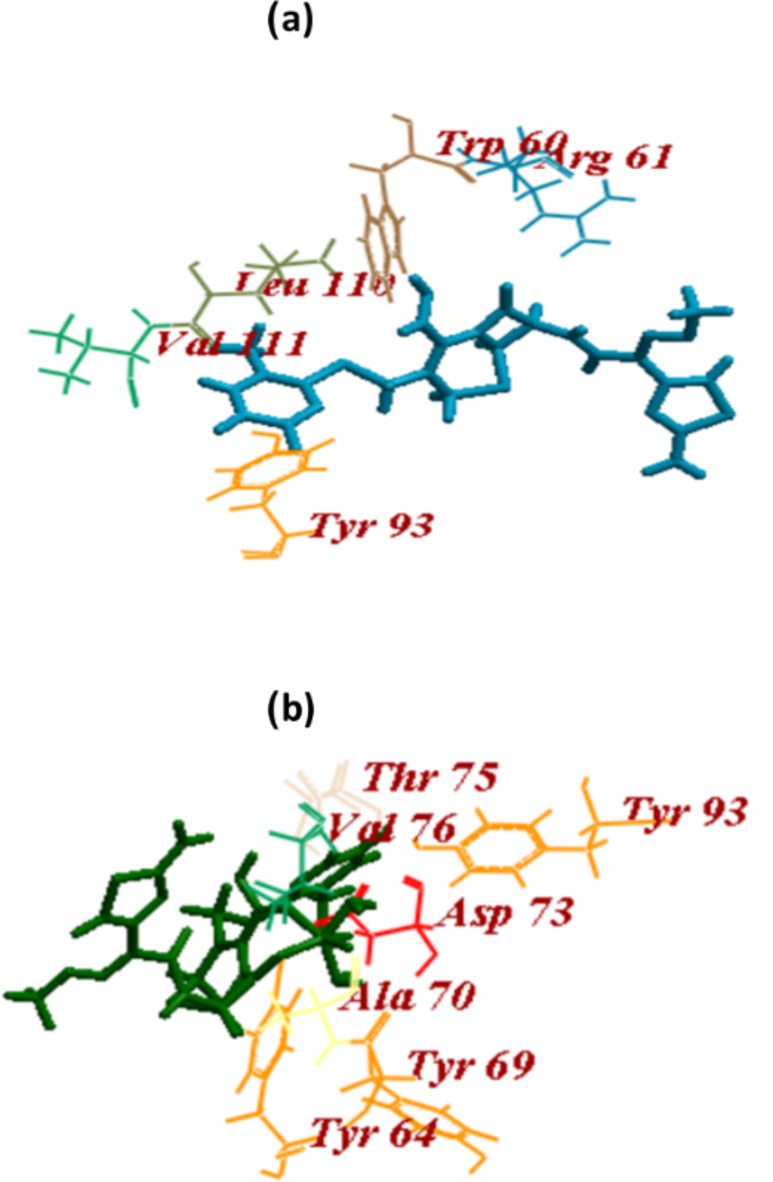
Docking of (a)ceftriaxone (CRO) and (b) ceftriaxone-Ni (CRON) into the binding site of LasR.

The autoinducer 3-oxo-C12-HSL was docked into the LasR binding site, this revealed three hydrogen bonds with amino acids, Ser129, Trp60, and Asp73, and an ICM score of − 107.47. CRO and CRON revealed ICM scores and H-bonds of -94.26 (9 H-bonds) and − 83.32 (21 H-bonds), respectively. CRO formed 2 H-bonds with Trp60, 2 H-bonds with Arg61, 3 H-bonds with Tyr93, 1 H-bond with Leu110 and another one with Val111. While CRON formed 4 H-bonds with Tyr64, 5 H-bonds with each Asp73 and Thr75, 3 H-bonds with Val76, 2 H-bonds with Tyr69 and 1 H-bond with both Ala70 and Tyr93 (Table 2; Fig. 6).

#### LasI and ligand binding affinity analysis

**Fig. 7:**
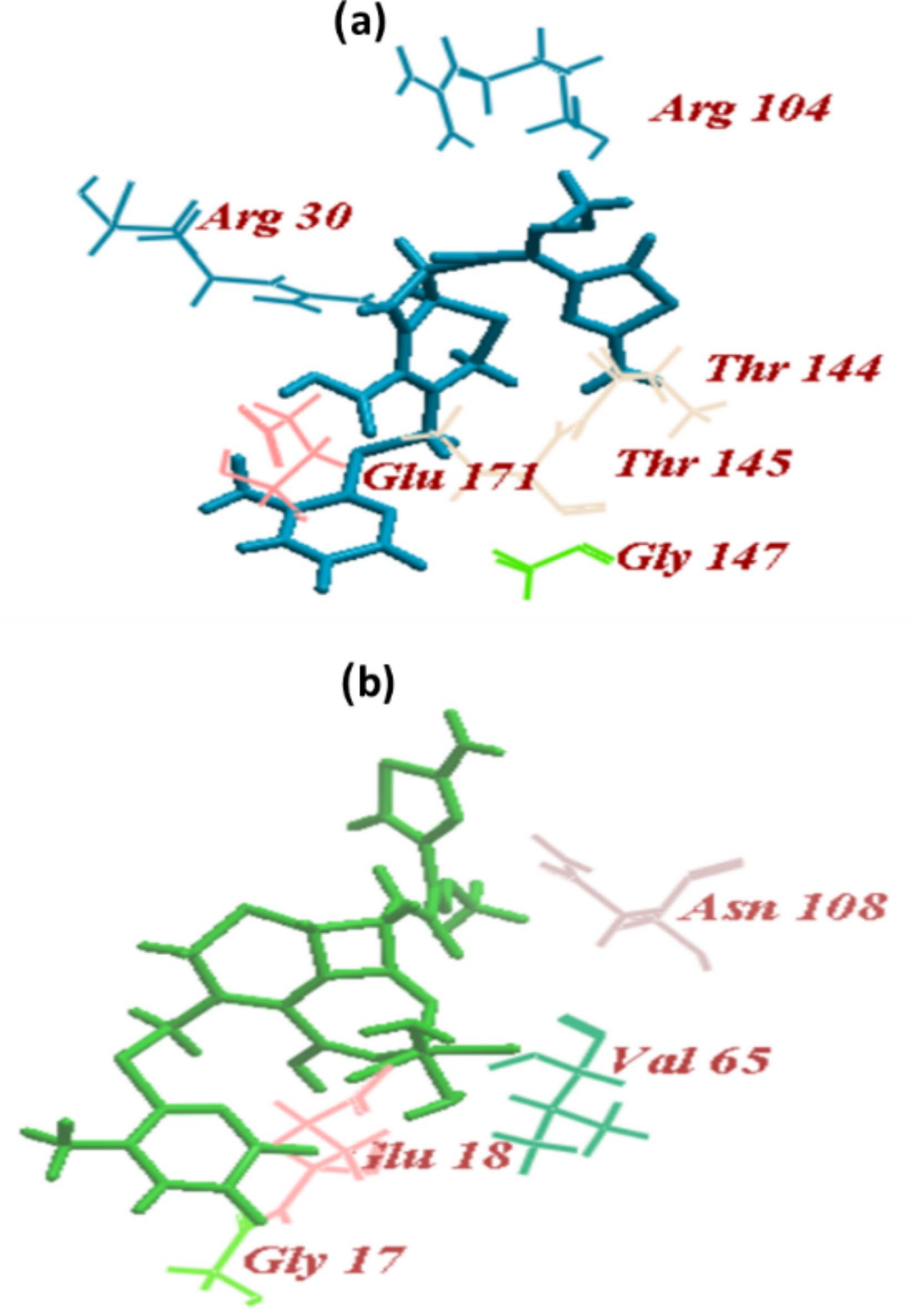
Docking of (a)ceftriaxone (CRO) and (b) ceftriaxone-Ni (CRON) into the binding site of LasI.

The original ligand, sulfate, was redocked at the LasI binding site, which had 12 hydrogen bonds with Arg30 and an ICM score of − 44.03. CRO and CRON revealed ICM scores of − 88.99 and − 58.49, respectively, which are higher than sulfate. CRO formed 15 H-bonds; 6 H-bonds with Arg30, 2 H-bonds with each of Arg104, and Glu171, 3 H-bonds with Thr144 and 1 H-bond with each of Thr145nand Gly147. On the other hand, CRON formed 11 H-bonds; 7 H-bonds with Glu18, 1 H-bond with each of Gly17 and Asn108, and 2 H-bonds with Val65 (Table 3; Fig. 7).

#### PqsR and ligand binding affinity analysis

**Fig. 8:**
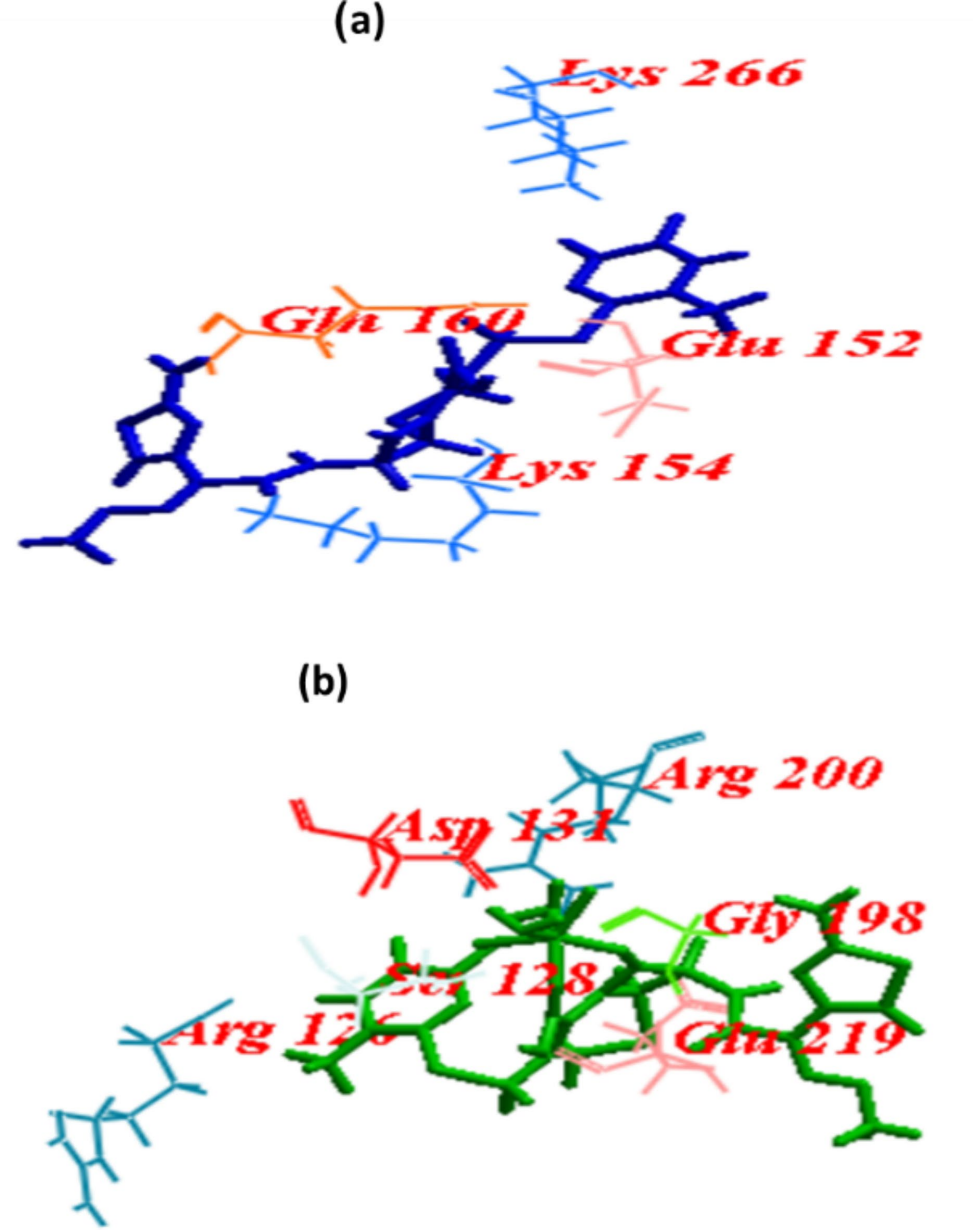
Docking of (a)ceftriaxone (CRO) and (b) ceftriaxone-Ni (CRON) into the binding site of PqsR.

NHQ, when redocked into the active site of the PqsR binding region revealed an ICM value of – 57.78 whereas CRO and CRON revealed ICM scores of − 75.93 and − 59.98 which are more than and comparable with NHQ respectively. CRO formed 12 H-bonds, 4 H-bonds with each of Lys154 and Gln160, 3 H-bonds with Lys266 and 1 H-bond with Glu152. Whereas, CRON formed 15 H-bonds, 5 H-bonds with Glu219, 3 H-bonds with each of Asp131 and Arg200, 2 H-bonds with Ser128 and 1 H-bonds with each of Arg126 and Gly198 (Table 4; Fig. 8).

## Discussion

*P. aeruginosa* stands out as a widespread opportunistic pathogen, renowned for its remarkable adaptability to diverse environmental conditions. QS serves as a crucial mechanism governing the expression of genes and virulence factors such as hemolysin, proteases, pyocyanin pigment, and biofilms. Unfortunately, the escalating prevalence of drug-resistant strains of *P. aeruginosa* has posed considerable challenges to effective treatment strategies recently. Therefore, it is essential to explore novel therapeutic techniques, one of which is to reduce *Pseudomonas* virulence factors by targeting the QS cascade with inhibitors. So, there is no need for concern about microbial resistance because the immune system can effectively eradicate bacterial infection. As far as we know, nothing is known about the QSI properties of CRON, and less is known regarding CRO QSI activity. In this work, *C. violaceum* ATCC 12,472 was used as a reporter strain to assess the QSI capabilities of CRO and its derivatives .*C. violaceum* ATCC 12,472 generates a number of N-acyl-L-homoserine lactones (AHLs), including N-(3-hydroxyundecanoyl)-homoserine lactone, N-(3-hydroxydecanoyl)-homoserine lactone, N-undecanoyl-homoserine lactone, N-nonanoyl-homoserine lactone, N-(3-oxodecanoyl)-homoserine lactone, L-homoserine lactone, and 3-oxo-C12-HSL. The primary inducer that regulates violacein synthesis through QS is N-(3-hydroxydecanoyl)-L-homoserine lactone. In the presence of additional signaling molecules such as 3-oxo-C10-HSL and 3-oxo-C12-HSL, *C. violaceum* ATCC 12,472 can produce more violacein. In this study, the production of violacein pigment was suppressed by CRO and CRON without influencing the growth of bacteria.

Remarkably, QS-related virulence factors of *P. aeruginosa* were decreased by CRO and CRON without affecting cell viability. QS plays a crucial role in *P. aeruginosa* pathogenesis by controlling the synthesis of many extracellular virulence factors and promoting the formation of biofilms^25^. Furthermore, *P. aeruginosa* exhibits a notable ability to form biofilm. Most traditional antibiotic regimens do not work on *Pseudomonas* biofilms. Reduction of biofilm formation increases the bacterial susceptibility to the immune system and neutrophil phagocytosis^26^. At 1/2 and 1/4 MICs, CRO and CRON demonstrated a notable decrease in biofilm formation (Fig. 2). Similarly, some other antibiotics could interfere with the QS cascade and reduce the production of biofilms. For example, imipenem^21^, azithromycin^27^, and tazobactam/piperacillin^22^. Moreover, *P. aeruginosa* biofilms become susceptible to tobramycin and phagocytosis due to the QSI activity of garlic^28^.

Additionally, *P. aeruginosa* produces the green pyocyanin pigment using PQS and rhlI/R signaling pathways^29^. CRO lowered the pyocyanin production among all studied strains of *P. aeruginosa*, including standard strains and clinical isolates at MICs of 1/2 and 1/4. Similarly, CRON significantly reduced pyocyanin production (Fig. 5). Likewise, juglone from walnuts^30^, carotenoid zeaxanthin^31^, luteolin, curcumin and apigenin^32^, and citrinin^33^ could reduce the production of *P. aeruginosa* virulence factors through QSI activity. Similarly, previous reports have demonstrated the QSI action of some antimicrobial drugs, such as azithromycin^34^, ceftazidime, tobramycin, ciprofloxacin, and cefoperazone^35,36^.

The transcription levels of QS genes were extremely low and could not be accurately assessed, supporting the idea that the investigated chemicals inhibit QS during the early transcriptional stages. This finding lends support to the idea that CRON may interfere with the transcriptional activation of virulence factors, hence inhibiting *P. aeruginosa* pathogenicity.

Additionally, CRO and CRON suppressed the las-regulated virulence factors, including protease. These results are in line with other β-lactams results including ceftazidime, cefepime, imipenem, and cefoperazone as they inhibited protease, pyocyanin, hemolysin, and biofilm production in *P. aeruginosa* standard and clinical strains without affecting the viability of cells^21–23^. Similarly, hemolysin production which is a las-regulated virulence factor was inhibited by CRO and CRON. These results are consistent with other studies which have shown that zinc oxide nanoparticles^37^ and metformin^38^ reduced the *P. aeruginosa*’s hemolytic activity.

In-silico experiments and virtual screening against LasR, LasI, and PqsR receptors were carried out to ascertain the QS inhibitory capacity of both CRO and CRON. The PDB structure of the receptor proteins LasI (PDB ID: 1RO5), LasR (PDB ID: 2UV0), and PqsR (PDB ID: 4JVD) was obtained through the Protein Data Bank. The binding mechanism, affinity, and orientation of the compounds were determined by computing the hydrogen bonds and scoring with the amino acids at the LasR active site. PDBsum indicated that the amino acids Ala50, Ala105, Cys79, Trp60, Trp88, Tyr93, Tyr56, Leu110, Gly126, Tyr64, Ser129, Asp73, and Thr75 were present at the LasR active site. The proper folding of the LuxR protein type is attributed to the formation of H-bonds between the residues Ser129, Trp60, and Asp73 of LasR and the polar groups of AHL^39–41^.

It has been revealed that oxo-C12-HSL and the amino acids Trp60, Asp73, and Tyr56 can form three H-bonds^42^. Numerous H-bonds, including Tyr56, Trp60, Arg61, Tyr64, Asp73, and Thr75, Val76, Tyr93, and Leu110, are formed between LasR and the two examined compounds in accordance with the CRO and CRON docking results. These findings are comparable to those on pyridoxal lactohydrazone^43^ and confirm that the tested compounds have interacted as the autoinducer and at the same binding site which is subsequently reflected in its high activity.

The LasI crystal structure showed that the residues Met125, Leu140, Met151, Phe105, Leu122, Trp69, Thr142, Leu157, Met79, Leu102, Leu188, Thr121, Val148, Met152, Ala155, Thr144, Ile178, and Thr121 formed a tunnel in the acyl-chain binding area. Among the residues that are well-conserved in AHL synthases are Thr142, Met79, Phe105, and Thr144^44^. Sulfate, the ligand of the enzyme interacted with Arg30 whereas CRO interacted with Gly147, Thr144, Arg30, Arg104, Thr145, and Glu171, most of them are well-conserved amino acids. On the other hand, CRON formed Gly17, Glu18, Val65, and Asn108. An ICM score of -57.78 was recorded for NHQ, which was redocked into the PqsR and established H-bonds with the amino acids in the PqsR enzyme’s active sites Leu208, Gln194, and Ile236^45^. When CRON and CRO were docked into PqsR’s active site, their ICM scores were − 59.98 and − 75.93, respectively. They bonded to amino acids Lys154, Gln160, Lys266, Gln160, Glu152, Ser128, Arg200, Glu219, Arg126, Asp131, Gly198 to generate H-bonds. The activity may be caused by its binding with LasR and LasI rather than PqsR because none of these were identified as amino acids at the active site.

This study added new insights to the growing research on quorum-sensing inhibition (QSI), by focusing on ceftriaxone (CRO) and its derivative, the CRO-Nickel complex. While earlier research has shown that antibiotics like imipenem, doxycycline, and ceftazidime exhibit QSI activity, with the main focus on single-component antibiotics and how they affect well-known quorum-sensing (QS) pathways, including LasR and RhlR systems. On the other hand, this research expands the range of possible quorum-sensing pathways impacted by investigating the involvement of CRON and CRO in targeting numerous QS receptors, such as LasR, LasI, and PqsR. Additionally, molecular docking data show that CRON and CRO have distinct binding affinities and distinct binding patterns with these QS receptors compared to other antibiotics. The improved QSI activity of the CRO-Nickel complex (CRON) was attributed to its unique hydrogen-bonding configurations and metal-coordination characteristics. The method by which CRO and its derivatives suppress quorum-sensing and virulence factors in *P. aeruginosa* is unique and has not been documented in earlier cephalosporin research. This work has important therapeutic implications. A possible new strategy for interfering with quorum-sensing pathways is the use of metal-drug complexes, such CRON, with substantial QSI effect. In the case of persistent *P. aeruginosa* infections, where biofilm formation is a major contributor to antibiotic resistance, this strategy might be especially helpful.

## Materials and methods

### Bacterial strains and growth media

The CRO and its metallic complexes were screened for their QSI activity using the reporter strain *Chromobacterium violaceum* ATCC 12,472^46^. This strain was cultivated overnight at 28 °C for 48 h after inoculation on Luria-Bertani (LB) media containing 0.5% (w/v) yeast extract, 1% (w/v) tryptone, and 1% (w/v) NaCl at pH 7. The media were solidified with 2% (w/v) agar^47^. Clinical isolates of *P. aeruginosa*, namely Pn11, Pn13, Pn15, and Pn17, were isolated from urine samples. The study was conducted in adherence to the ethical standards set by Alexandria University, Egypt. Informed consent was obtained from all participants who provided urine samples, and the study received ethical approval from the Alexandria University Ethics Committee (Approval No. 0201472, dated 18th March 2021). All procedures were performed in compliance with institutional and national guidelines for ethical research. The identification of *P. aeruginosa* strains was conducted using laboratory biochemical standards^48^. Standard strains *P. aeruginosa* PA14 and *P. aeruginosa* PAO1 were generously provided by Prof. Keller from the University of Washington, USA^49^. Additionally, Prof. Martin Schuster from the Department of Microbiology, Oregon State University, Nash Hall, Corvallis, OR 97,331, kindly supplied the QS-negative control strain *P. aeruginosa* PAO-JP2^50^. Each *P. aeruginosa* strain was cultured in LB broth medium and incubated overnight at 37 °C.

### Ceftriaxone and metallic derivatives

Following the procedure outlined by Masoud and colleagues^24^, CRO complexes with the transition metals: NiCl_2_ (CRON), ZnCl_2_ (CROZ), CuCl_2_ (CROU), and CdCl_2_ (CROD) (Sigma Aldrich, USA) were synthesized. The preparation of four basic metal-ceftriaxone complexes was carried out similarly. The inorganic salts [Ni(II), Zn(II), Cu(II), and Cd(II) as chlorides] were dissolved in 10 mL of bidistilled water. Two different mole ratios (M: L); 1:1 and 2:1, were utilized, combining the molar amount of the metal chloride salt with the estimated amount of the ligand. The reaction mixture was refluxed for five minutes and then left overnight. The resulting precipitated complexes were separated by filtration, washed repeatedly with a combination of ethanol and water, and finally dried in a vacuum desiccator over anhydrous CaCl_2_^24^.

### Reporter strain assay of potential inhibitors

To evaluate the QSI activity of CRO and the prepared compounds, the reporter strain *C. violaceum* ATCC 12,472 was used. Cultures of *C. violaceum* were incubated in a shaking incubator at 28 °C with 200 rpm agitation. A double-layer plate was employed using a layer of LB media; 2% (w/v) agar was poured onto a soft layer of LB medium; 1% (w/v) agar, was inoculated with 0.1 mL of *C. violaceum*. Wells with a diameter of 10 mm were created in the agar using a cork borer. Each well was loaded with 0.1 mL of CRO and tested compounds. Subsequently, the plates were then incubated, and after 24 h, the diameter of the violacein pigment discoloration zone was measured^46,51^. As a control, DMSO was added to one well in each plate. The compounds which showed the elimination of violacein pigment will be selected for the next steps.

### Minimum inhibitory concentration

To determine the minimum inhibitory concentrations (MICs) of CRO and CRON, the microtiter plate assay method was employed. Each well of the microtiter plate was filled with 0.1 mL of Muller-Hinton broth. The first well was loaded with 0.1 mL of the compound under evaluation. In the following wells, the tested substance was serially diluted twice, yielding serial dilutions of (512 to 0.5) µg/mL. A volume of 1.0 × 10^5^ CFU of the *P. aeruginosa* cultures, PA14, PAO1, Pn11, Pn13, Pn15, and Pn17 was added to each well. Each plate included positive and negative controls. After an overnight incubation period at 37 °C, microbial growth was observed on each plate. The MIC was determined as the lowest concentration of the compound at which microbial growth was completely inhibited, compared to the positive control^52^. Sub-MICs for CRO and CRON were also determined, and the strains under investigation were cultured in the presence of these sub-MICs.

### Sub-MIC impact on the growth of bacteria

*P. aeruginosa* strains PA14, PAO1, Pn11, Pn13, Pn15, and Pn17 were cultured with 1/2 MICs of CRO and CRON, following identical conditions applied for the cultivation of control untreated strains. Briefly, PA14, PAO1, Pn11, Pn13, Pn15, and Pn17 cultures were added to 25 mL of LB broth media containing 1/2 MIC of CRO and CRON, to obtain 0.05 OD600 nm at zero time. Also, untreated cultures were inoculated and grown under the same conditions. A volume of 1 mL of each mixture was taken at various intervals, and the optical density at 600 nm was calculated. Additionally, viable cell counts of untreated and treated *P. aeruginosa* strains PA14, PAO1, Pn11, Pn13, Pn15, and Pn17 were determined. For inoculation, 15 mL of LB agar was evenly distributed on a 9 cm plate, and 1 mL of each dilution was plated onto it. Samples were diluted 1:10, solidified, and incubated at 37 °C overnight. Bacterial counts were calculated as CFU/mL by multiplying viable colonies of *P. aeruginosa* by the dilution factor^53^.

### Antivirulence effect of CRO and CRON on *P. Aeruginosa* virulence factors

The virulence factors of *P. aeruginosa* strains were measured following treatment with CRO and CRON at 1/2 and 1/4 MICs in triplicate. Virulence variables were assessed both with and without the presence of CRO and CRON^21^. Similarly, strains PAO1 and PA14 served as positive controls and they were tested under the same conditions^54^.

### Inhibition of biofilm assay

The biofilm formation of *P. aeruginosa* strains was evaluated using the microtiter plate. After adding 0.1 mL of treated (1/2 and 1/4 MICs of CRO and CRON) and untreated cultures to each well, plates were incubated at 37 °C for 24 h. Subsequently, the wells were washed with saline and then immersed in 0.15 mL of 100% methanol for 15 min to fix the bacterial cells. Bacterial cells were stained with 1% w/v crystal violet. Excess stain was removed by rinsing the plate with water, followed by air drying. To solubilize the dye from the biofilm, 0.15 mL of 33% (v/v) glacial acetic acid was added to each well. The absorbance was measured at OD490^55^ and the percentage inhibition of biofilm by potential inhibitors was determined compared to untreated cells.

### Assay hemolysin

To perform the hemolysin test, supernatants from *P. aeruginosa* cultures from both treated (1/2 and 1/4 MICs of CRO and CRON) and untreated samples were mixed with rinsed red blood cells (RBCs) suspension^56^. A volume of 0.7 mL of erythrocytes was combined with 0.5 mL of cell-free supernatant. After two hours of incubation at 37 °C, the mixture was centrifuged for ten minutes at 4 °C at 4000 rpm. The degree of hemolysin activity in the supernatant was determined by measuring its absorbance at OD540 nm^57^.

### Assay of protease

The skimmed milk method was used to evaluate total protease production in *P. aeruginosa* cultures, both with and without sub-MICs of CRO and CRON^36^. In this experiment, 1 mL of 1.25% (w/v) skim milk was combined with 0.5 mL of supernatants from the *P. aeruginosa* cultures. After incubating the mixture for one hour, the absorbance was measured at OD600 nm. A reduction in OD600 indicates an increase in proteolysis activity, indicating that the skim milk has been degraded^58^.

### Inhibition of pyocyanin assay

Pyocyanin pigment synthesis in *P. aeruginosa* strains was evaluated by cultivating the tested isolates in King A broth media containing peptone (2% w/v), MgCl_2_ (0.14% w/v), and K_2_SO_4_ (1.0% w/v), with or without sub-MICs of CRO and CRON. The strains were cultured at 37 °C for 48 h with agitation at 200 rpm. To extract pyocyanin from the cultures, chloroform was added to the culture broth. The mixture was vortexed until a greenish-blue color developed, indicating the presence of pyocyanin. Subsequently, the mixture was centrifuged at 3000 rpm for 10 min to separate the chloroform layer. Following separation, 1 mL of 0.2 M HCl was added to the chloroform layer until the liquid turned pink. The absorbance of the solution was then measured at OD520 nm. To determine the pyocyanin concentration in µg/mL, the absorbance at OD520 nm was multiplied by 17.072^59,60^.

### Expression of QS genes

The expression of QS regulatory genes (lasI, lasR, rhlI, and rhlR) in *P. aeruginosa* PAO1 was evaluated using RT-PCR to assess the impact of the tested chemical; CRON. Cultures treated with 1/2 MIC of CRON and the untreated control were grown to an OD 600 nm of 0.4–0.5. Cells were collected by centrifugation for 15 min at 6000 × g. RNA was extracted using TRIZOL reagent (Oxoid, Basingstoke, Hants, UK) according to the manufacturer’s protocol, and complementary DNA (cDNA) was synthesized using the SensiFAST™ cDNA Synthesis Kit (Bioline Reagents Ltd., London, UK). RT-PCR was performed using the Rotor-Gene Q thermocycler (Qiagen, Valencia, CA, USA). The amplification reaction was carried out using TOPreal™ qPCR 2 × PreMIX (SYBR Green with low ROX) (Enzynomics; Daejeon, Korea) and the primers listed in Supplementary Table **S1**. Gene expression was normalized to the housekeeping gene rpoD to determine relative expression. The relative gene expression levels in CRON treated cultures were compared to control cultures using the 2^−ΔΔCT^ formula^61^.

### Molecular docking study

To explore the binding interactions of CRO and CRON with three QS systems in *P. aeruginos*a, namely LasI, LasR, and PqsR, a molecular docking study was conducted. Crystal structures of these QS systems were obtained from the Protein Data Bank: LasI (PDB ID: 1RO5)^44^, LasR (PDB ID: 2UV0)^62^, and PqsR (PDB ID: 4JVD)^45^. The components of the structures were built using ChemBioDraw (PerkinElmer Informatics), and energy minimization was performed using MM2. Subsequently, the PDB file for LasR (PDB ID: 2UV0) was converted into an internal coordinate mechanics (ICM) object using Molsoft software, as described by^63^ ICM aims to compute the minimum energy of ligand-receptor interactions. For the docking experiments, the 3oxo-C12-HSL molecule associated with the LasR structure (PDB ID: 2UV0) was used as a common docked model of the autoinducer molecule. The molecular docking study aimed to assess the affinities, binding modes, and inhibitory activities of CRO and CRON with these QS systems in *P. aeruginosa*.

### Statistical analysis

The experiments were carried out in triplicate, and an Excel data sheet was used to calculate the means and standard deviations of the three distinct measurements. *P < 0.05, **P ≤ 0.01, or ***P ≤ 0.001 were the possible probability values, and differences between strains that were treated with or without CRO and CRON were deemed significant. Welch’s t-test was utilized to evaluate the statistical analysis.

## Conclusion

In this study, we examined the quorum-sensing inhibitory (QSI) activity of ceftriaxone (CRO) and its derivative, the CRO-Nickel complex (CRON), in *P. aeruginosa*. This research showed that CRO and CRON could reduce quorum-sensing-regulated virulence factors such as biofilm formation, hemolysin, and protease at sub-inhibitory concentrations without inhibiting bacterial growth. Herein, the molecular docking studies revealed potential interactions between these drugs and major quorum-sensing receptors (LasR, LasI, and PqsR), implying that these interactions may contribute to the reported QSI activity. The binding affinities elucidated via molecular docking studies require further validation by in vivo research and direct measurements of quorum-sensing molecules. These findings pave the way for further study, but they should not be taken as definitive proof of their superiority or clinical efficacy.

**Table 1:** MICs and sub-MICs of Cefotriaxone (CRO) and cefotriaxone-nickel complex (CRON).

	CRO			CRON		
	MIC µg/mL	1/2 MIC µg/mL	1/4 MIC µg/mL	MIC µg/mL	1/2 MIC µg/mL	1/4 MIC µg/mL
*P. aeruginosa* PA14	4	2	1	2	1	0.5
*P. aeruginosa* PAO1	4	2	1	2	1	0.5
*P. aeruginosa* Pn11	16	8	4	4	2	1
*P. aeruginosa* Pn13	32	16	8	16	8	4
*P. aeruginosa* Pn15	8	4	2	2	1	0.5
*P. aeruginosa* Pn17	8	4	2	32	16	8

**Table 2:** Molecular docking results of ceftriaxone (CRO) and Ceftriaxone-Ni complex (CRON) with interacting amino acids with LasR QS system of *P. Aeruginosa*.

Drug	ICM Score	H-Bond	a a’ residues	Atom of a a’	Atom of comp.	length Å
CRO	-94.26	9	Trp 60	He1	O7	2.66
			Trp 60	He1	O1	1.31
			Arg 61	He	O2	2.68
			Arg 61	Hh22	O2	2.29
			Tyr 93	Hh	O6	1.26
			Tyr 93	Hh	O5	1.69
			Tyr 93	Hh	N8	2.48
			Leu 110	O	H15	1.71
			Val 111	O	H15	2.71
CRON	-83.32	21	Tyr 64	Hh	O7	1.95
			Tyr 64	Hh	O8	2.36
			Tyr 64	Hh	O2	2.24
			Asp 73	Hh	O8	2.62
			Thr 75	Hh	O6	2.38
			Thr 75	Hh	O5	1.81
			Thr 75	Hg1	O5	0.48
			Thr 75	Hg2	N8	1.98
			Val 76	Hn	O5	2.71
			Val 76	Hn	O7	2.69
			Val 76	Hn	O9	1.84
			Tyr 64	Oh	H20	2.22
			Tyr 69	O	H19	1.44
			Tyr 69	O	H20	2.61
			Ala70	O	H22	2.54
			Asp 73	Od2	H21	2.37
			Asp 73	Od3	H20	2.39
			Asp 73	O	H21	1.79
			Asp 73	O	H22	1.94
			Thr 75	Og1	H2	1.77
			Tyr 93	Oh	H15	1.63

**Table 3:** Molecular docking results of ceftriaxone (CRO) and Ceftriaxone-Ni complex (CRON) with interacting amino acids with LasI QS system of *P. Aeruginosa*.

Drug	ICM Score	H-Bond	a a’ residues	Atom of a a’	Atom of comp.	length Å
CRO	-88.99	15	Arg 30	Hh12	O1	2.15
			Arg 30	Hh12	O2	2.18
			Arg 30	Hh21	O2	0.70
			Arg 30	Hh21	O3	2.62
			Arg 30	Hh22	O2	1.42
			Arg 30	Hh22	O3	1.94
			Arg 104	Hn	N3	2.67
			Arg 104	Hn	O4	2.39
			Thr 144	Hg1	N4	1.42
			Thr 145	Hg1	N8	2.29
			Gly 147	Hn	O5	1.88
			Thr 144	Og1	H13	1.15
			Thr 144	Og1	H14	1.23
			Glu 171	Oe1	H2	1.49
			Glu 171	Oe2	H2	2.61
CRON	-58.49	11	Gly 17	Hn	O6	2.63
			Glu 18	Hn	O6	0.77
			Glu 18	Hn	O5	2.74
			Val 65	Hn	O8	2.14
			Val 65	Hn	O9	2.57
			Asn 108	Hd22	N4	1.75
			Glu 18	Oe1	H19	1.49
			Glu 18	Oe1	H20	1.12
			Glu 18	Oe1	H22	2.22
			Glu 18	O	H2	2.12
			Glu 18	O	H21	2.69

**Table 4:** Molecular docking results of ceftriaxone (CRO) and Ceftriaxone-Ni complex (CRON) with interacting amino acids with PqsR of *P. Aeruginosa.*

Drug	ICM Score	H-Bond	a a’ residues	Atom of a a’	Atom of comp.	length Å
CRO	-75.93	12	Lys 154	Hn	O1	2.06
			Lys 154	Hn	O2	2.04
			Lys 154	Hz1	O3	1.29
			Lys 154	Hz3	O3	2.27
			Gln 160	He21	O7	2.40
			Gln 160	He22	O7	2.23
			Lys 266	Hz2	O6	2.22
			Lys 266	Hz2	O5	1.95
			Lys 266	Hz3	O5	2.45
			Glu 152	O	H2	1.58
			Gln 160	O	H13	1.48
			Gln 160	O	H14	2.74
CRON	-59.98	15	Ser 128	Hg	O7	2.65
			Ser 128	Hg	O9	1.92
			Arg 200	Hh12	O5	2.52
			Arg 200	Hh21	O6	2.21
			Arg 200	Hh21	O5	2.39
			Glu 219	Hn	O3	2.16
			Glu 219	Hn	N3	2.36
			Arg 126	O	H15	2.19
			Asp 131	Od2	H19	1.62
			Asp 131	Od2	H20	1.72
			Asp 131	Od2	H21	1.67
			Gly 198	O	H22	1.34
			Glu 219	Oe1	H8	1.34
			Glu 219	Oe2	H8	2.53
			Glu 219	O	H2	1.12

## Electronic supplementary material

Below is the link to the electronic supplementary material.

Supplementary Material 1

## Data Availability

The datasets used and analyzed in the current study are available from the corresponding author on reasonable request.
