# Supplementary material for: Innovative application of ceftriaxone as a quorum sensing inhibitor in Pseudomonas aeruginosa
Source: Sci Rep. 2025 Feb 11;15:5022. doi: 10.1038/s41598-025-87609-0 (PMC11814147; doi:10.1038/s41598-025-87609-0)
Supplement: Supplementary file 1 — Supplementary Material 1 [file 41598_2025_87609_MOESM1_ESM.docx]

**Innovative Application of Ceftriaxone as A Quorum Sensing Inhibitor in *Pseudomonas aeruginosa***

**Nourhan G. Naga^1^*, Dalia E. El-Badan^1,2^, Mona E. Mabrouk^3^, Heba S. Rateb^4^, Khaled M. Ghanem^1^, and Mona I. Shaaban^5^*******

^1^Department of Botany and Microbiology, Faculty of Science, Alexandria University, Egypt

^2^Department of Biological Sciences, Faculty of Science, Beirut Arab University, Beirut, Lebanon

^3^ Botany and Microbiology Department, Faculty of Science, Damanhour University, Damanhour, Egypt

^4^Department of Pharmaceutical and Medicinal Chemistry, Pharmacy College, Misr University for Science and Technology, Cairo, Egypt.

^5^Department of Microbiology and Immunology, Faculty of Pharmacy, Mansoura University, Egypt

| **Gene Type** | **Gene Name** | **Primer Type** | **Primer Sequence** | **Melting Temp. (°C)** | **Amplicon Size (bp)** |
| --- | --- | --- | --- | --- | --- |
| **Reference Gene** | rpoD | Fw | 5′–CGA ACT GCT TGC CGA CTT–3′ | 56°C | 131 |
|  |  | Rev | 5′–GCG AGA GCC TCA AGG ATA C–3′ |  |  |
| **QS Genes** | lasR | Fw | 5′–CTG TGG ATG CTC AAG GAC TAC–3′ | 55°C | 133 |
|  |  | Rev | 5′–AAC TGG TCT TGC CGA TGG–3′ |  |  |
|  | rhlI | Fw | 5′–GTA GCG GGT TTG CGG ATG–3′ | 58°C | 101 |
|  |  | Rev | 5′–CGG CAT CAG GTC TTC ATC G–3′ |  |  |

**Supplementary Table S1: Primer Sequences and Amplification Parameters Used for RT-PCR Analysis of QS Regulatory Genes in P aeruginosa**

**
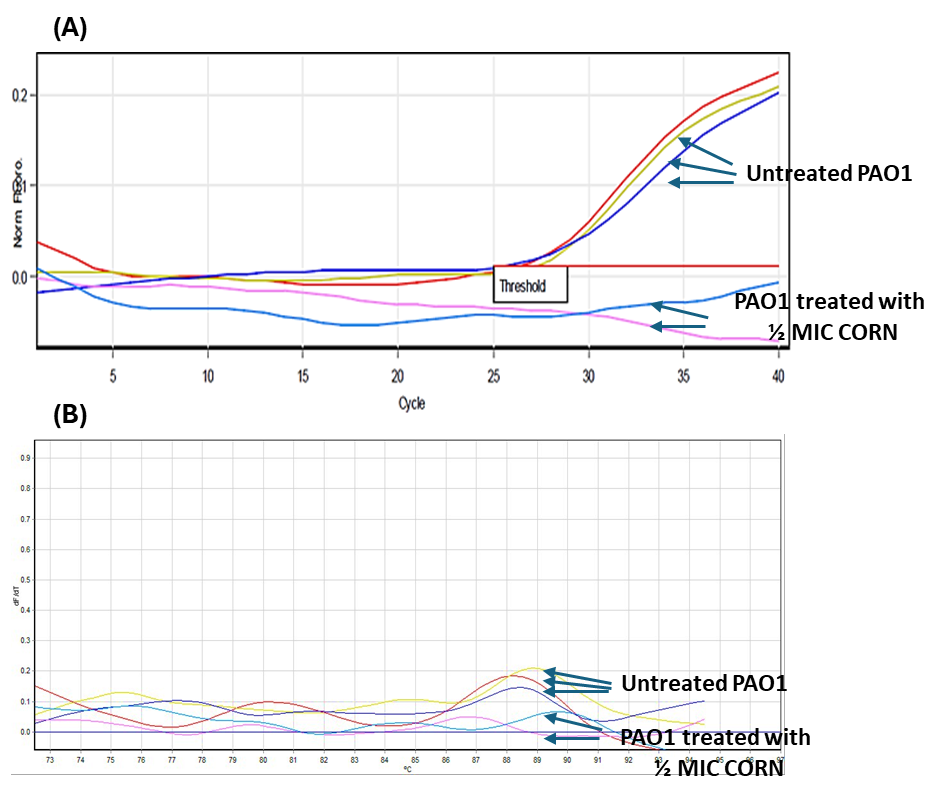
**

**Supplementary Fig. 1:** RT-PCR of *lasR* gene in *P.* *aeruginosa* PAO1, untreated and ceftriaxone Nickel complex (CRON) treated with A) Amplification plot, B) Melting curve.

**
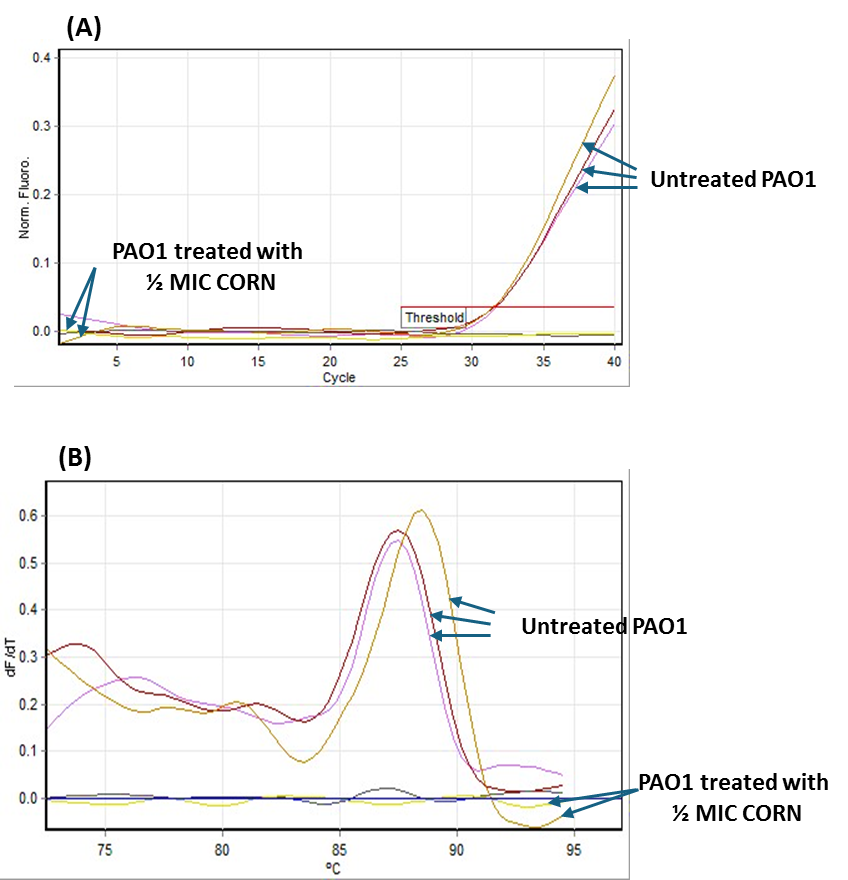
**

**Supplementary Fig. 2:** RT-PCR of *rhlR* gene in *P.* *aeruginosa* PAO1, untreated and ceftriaxone Nickel complex (CRON) treated with A) Amplification plot, B) Melting curve.
